# Propofol Ameliorates Microglia Activation by Targeting MicroRNA-221/222-IRF2 Axis

**DOI:** 10.1155/2021/3101146

**Published:** 2021-08-10

**Authors:** Xi Xiao, Yuanyuan Hou, Wei Yu, Sihua Qi

**Affiliations:** ^1^Department of Anesthesiology, The Fourth Affiliated Hospital of the Harbin Medical University, Harbin, 150001 Heilongjiang Province, China; ^2^Department of Anesthesiology, First Affiliated Hospital of Dalian Medical University, Dalian, 116011 Liaoning Province, China

## Abstract

**Background:**

Propofol is a widely used intravenous anesthetic drug with potential neuroprotective effect in diverse diseases of neuronal injuries such as traumatic brain injury and ischemic stroke. However, the underlying molecular mechanism remains largely unknown.

**Methods:**

Real-time qPCR, enzyme-linked immunosorbent assay, and Western blotting were used to identify the expression pattern of miR-221/222, inflammatory genes, cytokines, and IRF2. The biological roles and mechanisms of propofol in microglia activation were determined in BV2 cells and primary microglia. Bioinformatic analysis and luciferase reporter assay were used to confirm the regulatory role of miR-221/222 in Irf2 expression.

**Results:**

We found that miR-221 and miR-222 were downstream targets of propofol and were consistently upregulated in lipopolysaccharide- (LPS-) primed BV2 cells. Gain- and loss-of-function studies revealed that miR-221 and miR-222 were profoundly implicated in microglia activation. Then, interferon regulatory factor 2 (Irf2) was identified as a direct target gene of miR-221/222. IRF2 protein levels were reduced by miR-221/222 and increased by propofol treatment. Ectopic expression of IRF2 attenuated the proinflammatory roles induced by LPS in BV2 cells. More importantly, the suppressive effects of propofol on LPS-primed activation of BV2 cells or primary mouse microglia involved the inhibition of miR-221/222-IRF2 axis.

**Conclusions:**

Our study highlights the critical function of miR-221/222, which inhibited *Irf2* translation, in the anti-inflammatory effects of propofol, and provides a new perspective for the molecular mechanism of propofol-mediated neuroprotective effect.

## 1. Introduction

As the main neuroimmune cells of the brain, microglia play critical roles in maintaining normal brain function [1]. Under physiologic conditions, microglial cells maintain tissue homeostasis and contribute towards brain development [2]. In response to any type of pathologic events (injury, infection, and inflammation) or changes in brain homeostasis, microglia rapidly switch to an activated state, undergo substantial morphological, molecular, and functional changes, and produce a broad spectrum of proinflammatory mediators, such as nitric oxide (NO), reactive oxygen species (ROS), and proinflammatory cytokines, including interleukin 1beta (IL-1*β*), IL-6, and tumor necrosis factor-alpha (TNF-*α*), resulting in inflammation-induced neuronal cell damage or death [3]. The immortalized murine BV2 microglia have been widely used as an *in vitro* cell model to investigate the molecular mechanisms underlying microglial activation [4–6]. Emerging studies show that microglia activation has protective effects for neurons by attenuating neuronal apoptosis, increasing neurogenesis, and promoting functional recovery. In contrast, uncontrolled and overactivated microglia play a deleterious role and trigger neurotoxicity [7, 8]. Therefore, suppressing microglial activation bears great promise as an effective therapeutic strategy to mitigate neuronal damage and treat neurodegenerative diseases [9].

Propofol (2,6-diisopropylphenol) is a short-acting, widely used intravenous anesthetic agent for the induction and maintenance of anesthesia and for sedation in the intensive care unit (ICU) [10–12]. Propofol can reduce cerebral oxygen consumption, decrease intracranial pressure, and has potent anticonvulsant properties, thus making it a suitable anesthetic agent for neurosurgical patients [13]. Propofol also exhibits neuroprotective effects in diverse animal models of neuronal injury, including ischemia-reperfusion and traumatic brain injury [14, 15]. Previously, accumulated studies have revealed that propofol exhibits anti-inflammatory and antioxidative effects by reducing the biosyntheses of TNF-*α*, interleukin 1beta (IL-1*β*), IL-6 and decreasing the expression of NO [16–19]. However, the detailed molecular mechanism responsible for the anti-inflammatory properties of propofol in microglia activation remains largely unclear.

MicroRNAs (miRNAs) are a class of endogenous, noncoding RNA molecules (typically 21 nt) that can regulate gene expression by imperfect base-pairing with the 3′-untranslated region (3′-UTR) of target mRNAs [20, 21]. Through binding to target mRNA, miRNAs lead to mRNA degradation and/or translational repression and are known to be involved in the pathogenesis of nervous system, such as neuroinflammation, Alzheimer's disease, and autoimmune diseases [22–24]. The cotranscribed miR-221/222 family was well documented to promote malignant phenotypes, including tumor growth, metastasis, chemotherapy resistance, and angiogenesis [25–29]. Notably, the miR-221/222 cluster is able to regulate the expression of p27/mTOR in Parkinson's disease [30]. Moreover, miR-221 and miR-222 also play an important role in neuronal proliferation and differentiation by targeting SOX2 and KLF4 [31]. However, the role of miR-221/222 in microglia activation remains to be elucidated.

In the present study, we identified that miR-221 and miR-222 are potential targets in the protective effects of propofol in LPS-induced neuroinflammation. Our results showed that propofol could suppress the production of proinflammatory mediators by downregulating the expression level of miR-221/222. In addition, we demonstrated that the anti-inflammatory transcriptional factor IRF2 is a novel target of miR-221 and miR-222, which directly bind to its 3′-UTR inhibiting its translation. Using the BV2 cell model and primary microglia cultures, we confirmed the roles of miR-221/222-IRF2 axis in the anti-inflammatory function of propofol in microglia activation.

## 2. Materials and Methods

### 2.1. Cell Culture and Reagents

The murine microglia BV2 cell line was obtained from the China Center for Type Culture Collection and tested for mycoplasma contamination. BV2 cells were cultured in Dulbecco's modified Eagle's medium (Gibco, Shanghai, China) supplemented with 10% (v/v) fetal bovine serum (FBS, Gibco, Shanghai, China), 100 U/ml penicillin, and 100 mg/ml streptomycin at 37°C in a humidified incubator with 5% CO_2_. Confluent cells were passaged by trypsinization and cultured with a complete medium overnight before treatments. Propofol (Sigma, St. Louis, MO, USA) and lipopolysaccharide (LPS, Sigma, St. Louis, MO, USA) were dissolved in DMSO to prepare a stock solution. The final concentration of DMSO was lower than 0.01% to avoid possible nonspecific effects.

### 2.2. Cell Transfection

The miR-221/222 mimics or inhibitors and their negative control duplex were all synthesized by GenePharma Inc. (Shanghai, China). Detailed sequence information for inhibitors was shown as follows: mmu-miR-221 inhibitors, 5′-GAAACCCAGCAGACAAUGUAGCU-3′ and mmu-miR-222 inhibitors, 5′-AGACCCAGUAGCCAGAUGUAGCU-3′. The miRNA Transfection X-treme GENE Reagent (Roche, USA) was used for transient transfection following the manufacturer's instructions. Transfection of BV2 cells or primary microglia with small interfering RNAs (siRNAs) against *Irf2* and control-siRNAs (GenePharma, Inc., Shanghai, China) was performed using the jetPRIME transfection reagent (114-15, Polyplus-transfection, France) according to the manufacturer's instructions. Briefly, BV2 cells at 70-80% confluence were prepared. Then, 50 nM siRNAs diluted with 200 *μ*l jetPRIME buffer and 4 *μ*l jetPRIME transfection reagents were mixed. Finally, the transfection mix was added to the cells in serum-containing medium and allowed to culture for an additional 24 h. Knockdown efficiency of Irf2 was verified by Western blotting. The siRNA information was shown as follows: si-Irf2-1, 5′-GCUCUACCCUCUACAGAUUTT-3′ and si-Irf2-2, 5′-GGCUGGAGGAGCAGAUAAATT-3′. Overexpression of Irf2 was performed by transfection of pcDNA3.1-IRF2 plasmids using Lipofectamine 2000 (Invitrogen, USA); empty control plasmids were used to generate control cells. After 8 h of transfection, cell culture medium was removed and replaced with a complete medium. Overexpression efficiency was verified by Western blotting.

### 2.3. Quantitative Real-Time PCR

Total RNA including miRNA was isolated using TRIzol reagent (Invitrogen, USA) according to the manufacturer's protocol. The RNA concentration and quality were determined by spectrophotometry using NanoDrop™ 2000 (Thermo Scientific, USA). For mRNA detection, 1 *μ*g of total RNA was reversely transcribed by primeScript RT Master kit (Takara Bio Inc., Japan). Quantitative real-time PCR reaction was performed with SYBR Green using the ViiA7 System (AB Applied Biosystems, USA). The primers used in this study were shown as follows: *Il1b* forward, 5′-GCAACTGTTCCTGAACTCAACT-3′; *Il1b* reverse, 5′-ATCTTTTGGGGTCCGTCAACT-3′; *Il6* forward, 5′-TAGTCCTTCCTACCCCAATTTCC-3′; *Il6* reverse, 5′-TTGGTCCTTAGCCACTCCTTC-3′; *Tnf* forward, 5′-CCCTCACACTCAGATCATCTTCT-3′; *Tnf* reverse, 5′-GCTACGACGTGGGCTACAG-3′; *Nos2* forward, 5′-GTTCTCAGCCCAACAATACAAGA-3′; *Nos2* reverse, 5′-GTGGACGGGTCGATGTCAC-3′; *Ptgs2* forward, 5′-TTCAACACACTCTATCACTGGC-3′; *Ptgs2* reverse, 5′-AGAAGCGTTTGCGGTACTCAT-3′; and *Actb* forward, 5′-GGCTGTATTCCCCTCCATCG-3′; *Actb* reverse, 5′-CCAGTTGGTAACAATGCCATGT-3′. MicroRNA real-time transcription-PCR quantification of miRNA expression was carried out using a Hairpin-it miRNA RT-PCR Quantitation Kit and TaqMan-microRNA assay kit (GenePharma, Suzhou, China) according to the manufacturer's protocol. U6 RNA was set as an internal control.

### 2.4. Enzyme-Linked Immunosorbent Assay (ELISA)

Levels of IL-1*β* (catalog number: MLB00C), IL-6 (catalog number: M6000B), and TNF-*α* (catalog number: MTA00B) in cell culture supernatants of BV2 or primary mouse microglia were detected using mouse sandwich ELISA kit (R&D Systems, Inc., USA) according to the manufacturer's instructions. Briefly, standards or testing samples were added to antibody-coated 96-well plates and incubated for 1 h at room temperature. After washing the plates three times, detection antibodies were added and allowed for incubation for another 1 h at room temperature. Plates were washed with washing buffer three times and incubated in horseradish peroxidase- (HRP-) conjugated streptavidin for 20 min at room temperature in dark. Substrate solution was added and allowed incubation for 30 min in the dark. Finally, stop solution was added to the plates, and absorbance at 450 nm was measured using a Multimode Microplate Reader (BioTEK, USA). A standard curve was used to calculate the levels of proinflammatory cytokine release, and the results were expressed in picogram per milliliter.

### 2.5. Western Blotting

Parental and transfected BV2 cells or primary microglia were washed with prechilled PBS and lysed in RIPA buffer (Sigma, Shanghai, China). Homogenates were clarified by centrifugation at 15,000 × g for 15 min at 4°C. Protein concentration was measured using BCA Protein assay kit (Pierce, Appleton, WI, USA). Protein samples were separated using 10% sodium dodecyl sulfate-polyacrylamide gels and transferred onto polyvinylidene difluoride (PVDF) membranes (Millipore, USA). After blocking with 5% (m/v) nonfat milk, the PVDF membranes were incubated with primary antibodies against IRF2 (12525-1-AP, Proteintech, dilution 1 : 1,000) at 4°C overnight. *β*-Actin (ab8227, Abcam, dilution 1 : 2,000) was used as an internal control. On the next day, the membranes were incubated with horseradish peroxidase- (HRP-) conjugated secondary antibody for 1 h at room temperature. After rinsing, the membranes were transferred into Bio-Rad ChemiDoc™ XRS system, and blots were generated using the ECL kit (Millipore, USA).

### 2.6. Luciferase Reporter Assay

The putative miR-221/222 complementary site in the 3′-UTR of *Irf2* mRNA or its mutant sequence was cloned into the p-MiR-reporter vector (Ambion, USA). For luciferase reporter assay, BV2 cells were seeded in 96-well plates and then cotransfected with p-MIR-reporter vectors with miR-221, miR-222 mimics, or control mimics using Lipofectamine 2000 (Invitrogen, USA). pRL-SV40 (Promega, WI, USA) was used as the control vector to balance transfection efficiency. After transfection for 48 h, the relative luciferase activity was detected using a dual-luciferase reporter assay system (Promega, USA).

### 2.7. Primary Microglia Cultures

Primary microglia cultures were prepared from cerebral cortices of C57BL/6J mice. Briefly, mice were sacrificed, and brains were carefully dissected, chopped, triturated, and freed from meninges in Hanks' balanced salt solution (Sigma-Aldrich, USA). Forebrains were gently minced, dissociated, resuspended in DMEM/F-12 medium (Gibco, Shanghai, China), and filtered by passing through a 70 mm cell strainer (Sigma). Cells were collected by centrifugation (1,000 × g, 10 minutes at 4°C) and resuspended in DMEM/F-12 containing 10% (v/v) fetal bovine serum (FBS, Gibco, Shanghai, China), 100 U/ml penicillin, and 100 mg/ml streptomycin and cultured on Poly-D-lysine coated 75 cm^2^ cell culture flask in a humidified incubator with 5% CO_2_ at 37°C. After 12-14 days in culture, floating microglia were harvested from mixed glia (astrocyte/microglia) cultures and reseeded into cell culture plates. On the next day, nonadherent cells were removed by changing the media, and after 1 h, cells were used for subsequent experiments.

### 2.8. Statistical Analysis

Data were presented as the means ± SD of at least three independent experiments. Statistical analysis was conducted with GraphPad Prism 5 (GraphPad Software, San Diego, CA, USA). The two-sided Student *t*-test or one-way ANOVA followed by Student-Newman-Keuls (SNK) test was used to compare data between groups. Differences were considered statistically significant when the *p* value was less than 0.05.

## 3. Results

### 3.1. miR-221 and miR-222 Are Downregulated by Propofol

**Figure 1:**
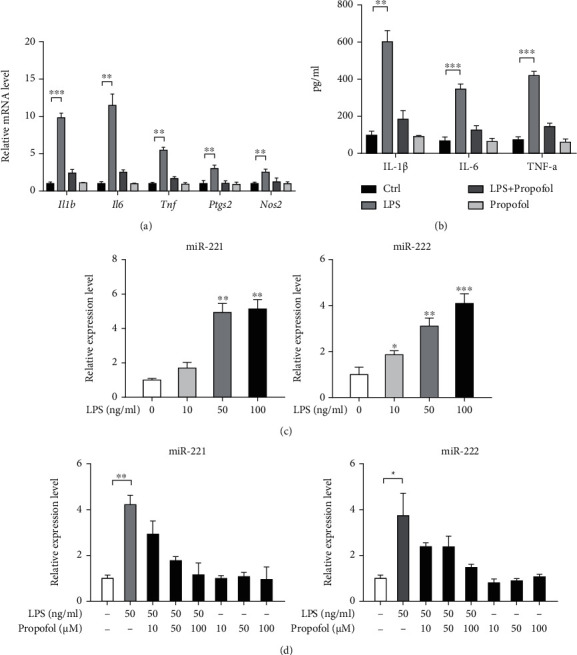
Expression of miR-221/222 is downregulated by propofol. (a) The inhibitory effects of propofol on the mRNA expression of inflammatory genes (*Il1b*, *IL6*, *Tnf*, *Ptgs2*, and *Nos2*) in LPS-primed BV2 cells were determined by real-time qPCR analysis (*n* = 3). (b) The inhibitory effects of propofol on the level of inflammatory cytokines (IL-1*β*, IL-6, and TNF-*α*) in conditioned medium from LPS-primed BV2 cells were analyzed by ELISA (*n* = 3). (c) miR-221 and miR-222 expression in BV2 cells upon treatment with different concentrations of LPS were analyzed by real-time qPCR (*n* = 3); comparisons were done with 0 ng/ml LPS. (d) miR-221 and miR-222 expression in LPS-primed BV2 cells upon treatment with different concentrations of propofol were analyzed by real-time qPCR (*n* = 3). ^∗^*p* < 0.05, ^∗∗^*p* < 0.01, and ^∗∗∗^*p* < 0.001.

Consistent with previous reports, propofol can significantly inhibit LPS-induced activation of BV2 cells as demonstrated by the expression of proinflammatory genes (*Il1b*, *IL6*, *Tnf*, *Ptgs2*, and *Nos2*) (Figure 1(a)) and secreted levels of proinflammatory cytokines (IL-1*β*, IL-6, and TNF-*α*) (Figure 1(b)). To determine the contribution of miR-221 and miR-222 in propofol-mediated anti-inflammatory effect in BV2 activation, we first detected their expression upon treatment with different concentrations of LPS (0, 10, 50, and 100 ng/ml). As a result, miR-221 and miR-222 expression were increased by LPS stimulation in a dose-dependent manner (Figure 1(c)). Importantly, LPS-induced upregulation of miR-221/222 can be largely compromised by the addition of propofol (Figure 1(d)), suggesting that propofol might affect miR-221/222 to hijack BV2 cell activation.

### 3.2. miR-221/222 Promotes Microglia Activation

**Figure 2:**
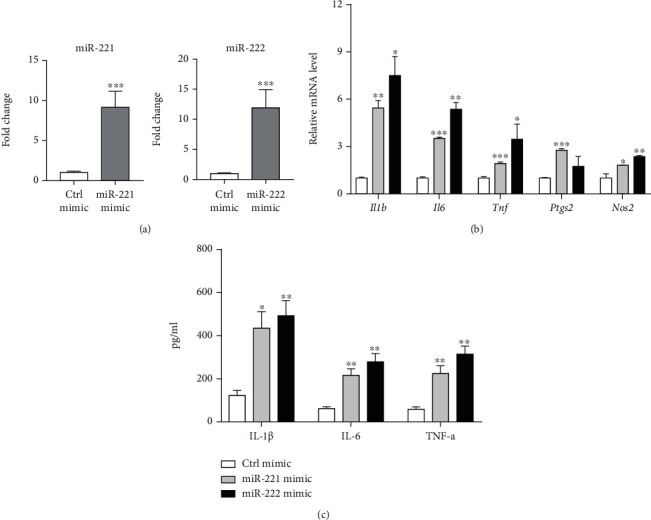
Effect of miR-221/222 on activation of BV2 microglia. (a) The transfection efficiency of miR-221 and miR-222 mimics was validated by qRT-PCR analysis. (b) The effects of miR-221 and miR-222 mimics on the mRNA expression of inflammatory genes (*Il1b*, *IL6*, *Tnf*, *Ptgs2*, and *Nos2*) in BV2 cells were determined by real-time qPCR analysis (*n* = 3). (c) The levels of inflammatory cytokines (IL-1*β*, IL-6, and TNF-*α*) in conditioned medium from BV2 cells upon treatment with miR-221 and miR-222 mimics were analyzed by ELISA (*n* = 3). ^∗^*p* < 0.05, ^∗∗^*p* < 0.01, and ^∗∗∗^*p* < 0.001; comparisons were done with ctrl mimic.

**Figure 3:**
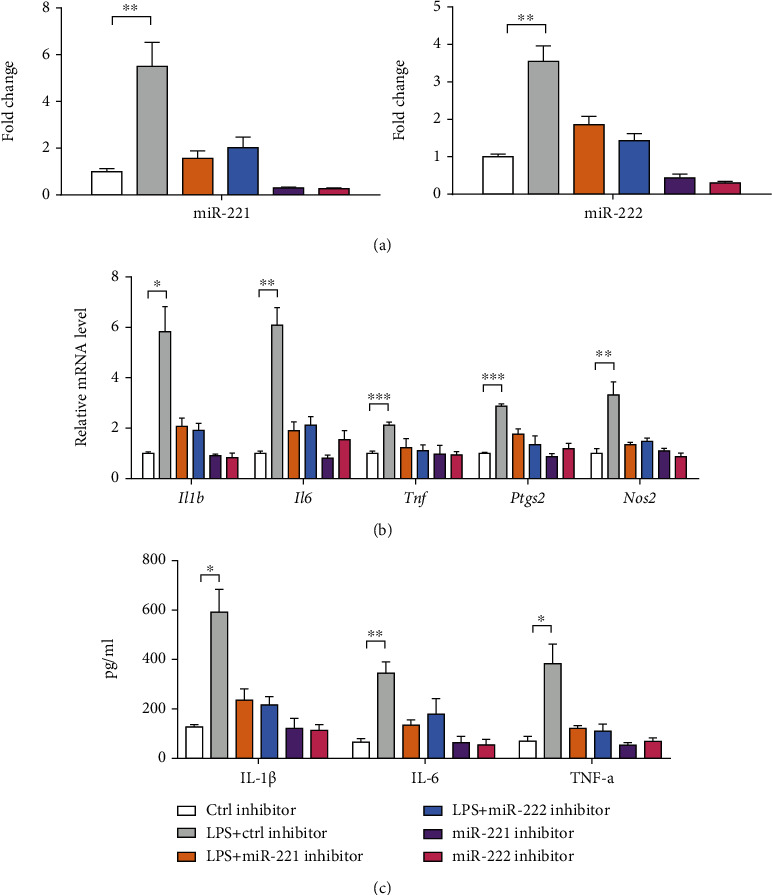
Inhibition of miR-221/222 blocks LPS-mediated BV2 activation. (a) The transfection efficiency of miR-221 and miR-222 inhibitors was validated by qRT-PCR analysis. (b) The effects of miR-221 and miR-222 inhibitors on the mRNA expression of inflammatory genes (*Il1b*, *IL6*, *Tnf*, *Ptgs2*, and *Nos2*) in LPS-primed BV2 cells were determined by real-time qPCR analysis (*n* = 3). (c) The effects of miR-221 and miR-222 inhibitors on the level of inflammatory cytokines (IL-1*β*, IL-6, and TNF-*α*) in conditioned medium from LPS-primed BV2 cells were analyzed by ELISA (*n* = 3). ^∗^*p* < 0.05, ^∗∗^*p* < 0.01, and ^∗∗∗^*p* < 0.001.

To investigate whether miR-221 and miR-222 are involved in microglia activation, we overexpressed miR-221/222 in BV2 cells by transfection with miR-221 and miR-222 mimics, respectively (Figure 2(a)). As shown in Figure 2(b), both miR-221 and miR-222 mimics promoted the mRNA expression of *Il1b*, *IL6*, *Tnf*, *Ptgs2*, and *Nos2*. In the cell culture supernatants, levels of IL-1*β*, IL-6, and TNF-*α* were also markedly increased by treatment with miR-221 or miR-222 mimics (Figure 2(c)). As the second line of evidence, we silenced miR-221/222 expression in LPS-primed BV2 cells by transfection with miR-221 and miR-222 inhibitors (Figure 3(a)). The results showed that LPS-induced upregulation of inflammatory genes (*Il1b*, *IL6*, *Tnf*, *Ptgs2*, and *Nos2*) (Figure 3(b)) and levels of inflammatory cytokines (Figure 3(c)) were remarkably attenuated by miR-221 or miR-222 inhibitors. Collectively, these findings support a regulatory role of miR-221/222 in facilitating microglia activation.

### 3.3. miR-221/222 Directly Targets IRF2

**Figure 4:**
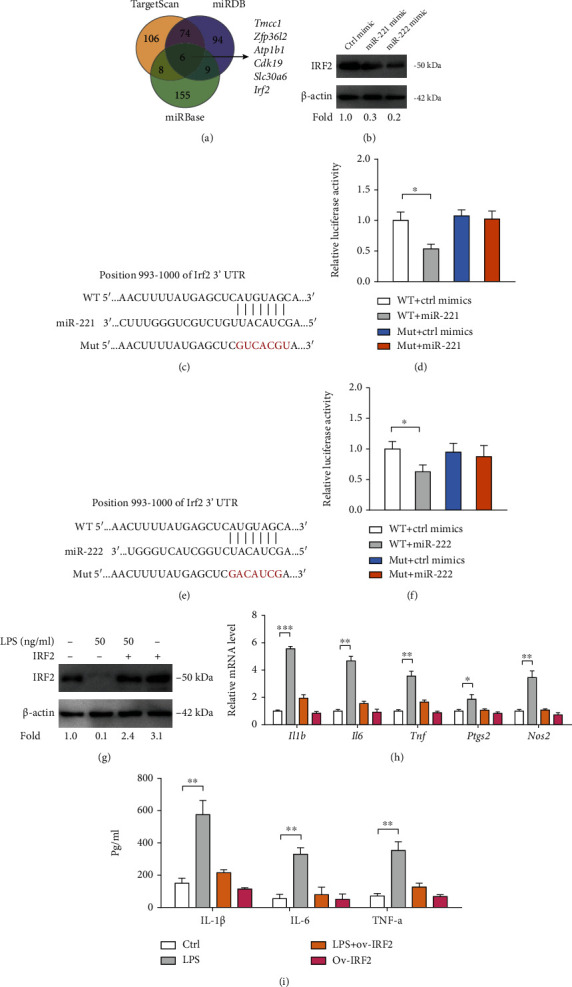
miR-221/222 directly targets IRF2. (a) Predictive targets of miR-221/222 from three independent databases (TargetScan, miRDB, and miRBase). (b) IRF2 protein expression in BV2 microglia after transfection of miR-221 and miR-222 mimics was analyzed by Western blotting; *β*-actin was loaded as a control. (c) Modeling miR-221 target sequences in 3′-UTR of IRF2; a mutant 3′-UTR with substitutions in the complementary site for the seed region of IRF2 was used. (d) BV2 cells were transfected with luciferase reporter vector (IRF2-WT or IRF2-Mut) and miR-221 mimics, and luciferase reporter activity was measured after transfection for 48 h. (e) Modeling miR-222 target sequences in 3′-UTR of IRF2; a mutant 3′-UTR with substitutions in the complementary site for the seed region of IRF2 was used. (f) BV2 cells were transfected with luciferase reporter vector (IRF2-WT or IRF2-Mut) and miR-222 mimics, and luciferase reporter activity was measured after transfection for 48 h. (g) IRF2 protein expression in BV2 microglia after LPS stimulation and/or transfection with pcDNA3.1-IRF2 was analyzed by Western blotting; *β*-actin was loaded as a control. (h) The effects of IRF2 overexpression on the mRNA expression of inflammatory genes (*Il1b*, *IL6*, *Tnf*, *Ptgs2*, and *Nos2*) in LPS-primed BV2 cells were determined by real-time qPCR analysis (*n* = 3). (i) The effects of IRF2 overexpression on the level of inflammatory cytokines (IL-1*β*, IL-6, and TNF-*α*) in conditioned medium from LPS-primed BV2 cells were analyzed by ELISA (*n* = 3). ^∗^*p* < 0.05, ^∗∗^*p* < 0.01, and ^∗∗∗^*p* < 0.001.

To decipher the downstream targets of miR-221/222 in microglia, three publicly available algorithms (TargetScan, miRDB, and miRBase) were used for prediction. The data revealed that six genes (*Tmcc1*, *Zfp36l2*, *Atp1b1*, *Cdk19*, *Slc30a6*, and *Irf2*) may be the targets of miR-221/222 (Figure 4(a)). Among these genes, the inflammation-related transcription factor gene, *Irf2*, was selected for further investigation. Indeed, ectopic expression of miR-221 or miR-222 led to an obvious reduction in IRF2 protein expression in BV2 cells (Figure 4(b)). To further test this possibility, we constructed a p-MIR-reporter containing the complementary seed sequence of miR-221/222 in the 3′-untranslated regions (3′-UTRs) of *Irf2* mRNA (WT) and a control reporter containing a mutated sequence (Mut) of the same fragment (Figures 4(c) and 4(e)). Then, reporter plasmid and miR-221/222 mimics were cotransfected into BV2 cells and subjected to luciferase reporter experiment. The result showed that miR-221/222 repressed the reporter activity driven by the 3′-UTRs of *Irf2* in BV2 cells; in contrast, luciferase activities were unaffected in the mutant form (Figures 4(d) and 4(f)). IRF2 protein level was remarkably downregulated by LPS stimulation (Figure 4(g)). Moreover, IRF2 overexpression blocked the LPS-induced expression of inflammatory genes and cytokines in BV2 cells (Figures 4(h) and 4(i)). Taken together, IRF2 might be a functional target of miR-221/222 in the activation of microglia.

### 3.4. Propofol Suppresses Microglia Activation via Targeting the miR-221/222-IRF2 Axis

**Figure 5:**
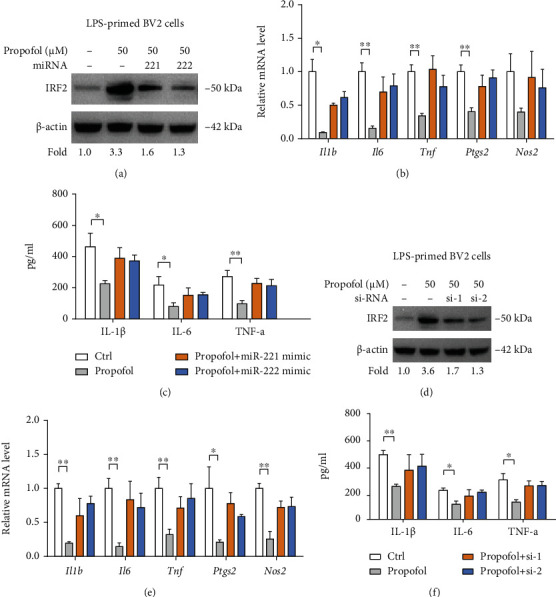
Propofol suppresses BV2 activation via targeting miR-221/222-IRF2 axis. (a) IRF2 protein expression in LPS-primed BV2 microglia upon treatment with propofol and/or miR-221/222 mimics was analyzed by Western blotting; *β*-actin was loaded as a control. (b) The effects of propofol and/or miR-221/222 mimics on the mRNA expression of inflammatory genes (*Il1b*, *IL6*, *Tnf*, *Ptgs2*, and *Nos2*) in LPS-primed BV2 cells were determined by real-time qPCR analysis (*n* = 3). (c) The effects of propofol and/or miR-221/222 mimics on the level of inflammatory cytokines (IL-1*β*, IL-6, and TNF-*α*) in conditioned medium from LPS-primed BV2 cells were analyzed by ELISA (*n* = 3). (d) IRF2 protein expression in LPS-primed BV2 microglia upon treatment with propofol and/or siRNAs against IRF2 was analyzed by Western blotting; *β*-actin was loaded as a control. (e) The effects of propofol and/or IRF2 siRNAs on the mRNA expression of inflammatory genes (*Il1b*, *IL6*, *Tnf*, *Ptgs2*, and *Nos2*) in LPS-primed BV2 cells were determined by real-time qPCR analysis (*n* = 3). (f) The effects of propofol and/or IRF2 siRNAs on the level of inflammatory cytokines (IL-1*β*, IL-6, and TNF-*α*) in conditioned medium from LPS-primed BV2 cells were analyzed by ELISA (*n* = 3). ^∗^*p* < 0.05 and ^∗∗^*p* < 0.01.

Next, we investigated whether the miR-221/222-IRF2 axis mediates the suppressive role of propofol in microglia activation (Figure 5(a)). To achieve this, we first overexpressed miR-221/222 in LPS-primed BV2 cells with propofol treatment. As displayed in Figure 5(b), propofol significantly inhibited the expression of inflammatory genes (*Il1b*, *IL6*, *Tnf*, *Ptgs2*, and *Nos2*), which can be restored by either miR-221 or miR-222 mimics. A similar result was observed as evidenced by inflammatory cytokines (Figure 5(c)). In addition, we genetically silenced IRF2 in LPS-primed BV2 cells with propofol treatment, and two siRNAs against *Irf2* had effective knockdown efficiency (Figure 5(d)). Expectedly, propofol failed to induce an inhibitory effect on BV2 cells with treatment of *Irf2* siRNAs (Figures 5(e) and 5(f)). Thus, the miR-221/222-IRF2 axis is an important functional mediator of propofol in suppressing microglia activation.

### 3.5. Propofol-miR-221/222-IRF2 Axis Is Involved in Primary Microglia Activation

**Figure 6:**
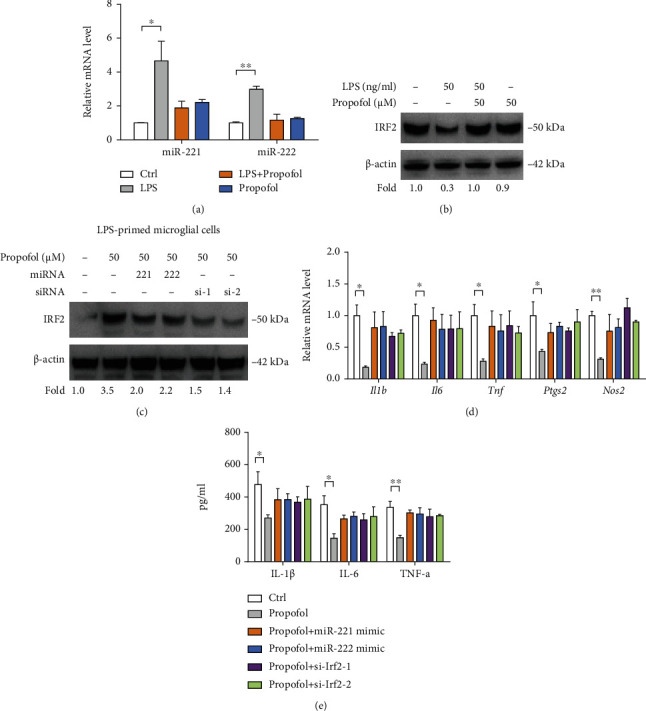
Propofol-miR-221/222-IRF2 axis is involved in primary microglia activation. (a) miR-221 and miR-222 expression in primary microglia upon treatment with LPS and/or propofol were analyzed by real-time qPCR (*n* = 3). (b) IRF2 protein expression in primary microglia upon treatment with LPS and/or propofol was analyzed by Western blotting; *β*-actin was loaded as a control. (c) IRF2 protein expression in LPS-primed primary microglia upon treatment with propofol, miR-221/222 mimics, and/or IRF2 siRNAs was analyzed by Western blotting; *β*-actin was loaded as a control. (d) The effects of propofol, miR-221/222 mimics, and/or IRF2 siRNAs on the mRNA expression of inflammatory genes (*Il1b*, *IL6*, *Tnf*, *Ptgs2*, and *Nos2*) in LPS-primed primary microglia were determined by real-time qPCR analysis (*n* = 3). (e) The effects of propofol, miR-221/222 mimics, and/or IRF2 siRNAs on the level of inflammatory cytokines (IL-1*β*, IL-6, and TNF-*α*) in conditioned medium from LPS-primed primary microglia were analyzed by ELISA (*n* = 3). ^∗^*p* < 0.05 and ^∗∗^*p* < 0.01.

To further confirm our findings above, we isolated primary microglia to investigate whether propofol has similar anti-inflammatory properties. After stimulation with 50 ng/ml LPS, primary microglia had a 5-fold increase of miR-221 and 3-fold increase of miR-222, and upregulated miR-221/222 expression can be suppressed by treatment with 50 *μ*M propofol (Figure 6(a)). Consistently, LPS-induced activation of primary microglia had reduced expression of IRF2 protein, which further can be increased by propofol treatment (Figure 6(b)). To determine the role of propofol and the miR-221/222-IRF2 axis in primary microglia activation, we overexpressed miR-221/222 or knocked down IRF2 in LPS-primed primary microglia with propofol treatment. As shown in Figure 6(c), miR-221/222 transfection or *Irf2* siRNAs robustly downregulated IRF2 protein expression. Propofol inhibited the expression of proinflammatory genes (*Il1b*, *IL6*, *Tnf*, *Ptgs2*, and *Nos2*) and levels of proinflammatory cytokines (IL-1*β*, IL-6, and TNF-*α*) in primary microglia; of note, these anti-inflammatory effects induced by propofol were abrogated by miR-221/222 transfection or addition of *Irf2* siRNAs in primary microglia (Figures 6(d) and 6(e)).

## 4. Discussion

Microglia activation is closely associated with the development and severity of several pathological diseases such as trauma, stroke, or chronic neurodegenerative. The present study identified that the miR-221/-222-IRF2 axis is involved in LPS-mediated microglia activation. Propofol can efficiently target the miR-221/-222-IRF2 axis to prevent microglial activation in two independent cell models.

Intense studies have revealed that propofol can regulate a plethora of genes related to the activation of microglia [19, 32–34]. Previously, we reported that propofol attenuates hypoxia-induced neuroinflammation, at least in part by inhibiting oxidative stress and NF-*κ*B/hypoxia inducible factor-1*α* (Hif-1*α*) signaling in BV2 microglia [35]. However, the knowledge of the roles of miRNAs on propofol-induced inactivation of microglia is limited [18]. In this study, we for the first time demonstrated a role of the clustered miR-221 and miR-222 in microglia activation with potential pathological relevance. miR-221/222 expression can be downregulated by propofol in a dose-dependent manner. Interestingly, propofol is sufficient to inhibit several inflammatory signaling pathways, such as NF-*κ*B, p38 MAPK, and JAK1/STAT3 pathway [35–37]. Thus, it will be interesting to investigate whether the inactivation of these inflammatory signaling pathways by propofol is responsible for the decrease in the expression of miR-221/222 in microglia.

Furthermore, we confirmed that the miR-221/222 cluster mediates the process of propofol-induced inactivation of microglia via targeting IRF2. IRF2 is originally identified as an inflammation-associated transcriptional repressor of the interferon-beta (IFN-beta) as well as of IFN-inducible genes and plays diverse roles in biological processes including oncogenic transformation, pathogen response, cytokine signaling, cell proliferation regulation, and hematopoietic development [38]. Under specific pathogen-free and steady-state conditions, IRF2 is expressed constitutively in a variety of cell types and tissues [39]. Consistent with this notion, we noticed that IRF2 protein level was significantly reduced in activated microglia and can be increased by propofol treatment. The reduced amount of IRF2 could at least in part explain propofol-induced inactivation of microglia, as it has been demonstrated that knockdown of IRF2 decreased IL-4-induced macrophage alternative activation [40]. Moreover, we found that the miR-221/222-IRF2 axis is a functional mediator of the anti-inflammatory roles of propofol. However, several other molecular targets of miR-221/222 have been reported, such as Notch3 [27], CACNA1C and KCNJ5 [41], PUMA and ETS-1 [42], and genes involved in inflammatory response (ETS1/2, IRF2, BCL2L11, TOX, BMF, and CXCL12) [43], and whether these targets are involved in the anti-inflammatory roles of propofol in microglia remains an area of active study. Notably, miRNA-based therapies for disease treatments are under rapid clinical development [24]; targeting mir-221/222 may provide innovative practical therapeutics for patients with traumatic brain injury and ischemic stroke.

There are also several limitations in the current study. Firstly, an *in vivo* model for traumatic brain injury or ischemic stroke will be better to interpret the neuroprotective effect of propofol via inhibition of microglia activation. Secondly, the downstream targets of IRF2 in BV2 cells or primary microglia were largely elusive, and whether IRF2 directly affects inflammatory genes via transcriptional repression is not known.

## 5. Conclusions

Our data show that propofol-induced downregulation of miR-221/222 resulted in the inactivation of BV2 microglia and primary microglia. Targeting miR-221/222 or ectopic expression of IRF2 significantly blocked microglial activation. Our findings might provide a novel molecular mechanism for the neuroprotective effects of propofol and suggested the importance of miRNA-based therapeutics in future treatment strategies for central nervous system injuries.

## Data Availability

All data generated or analyzed during this study are included in this manuscript.
